# Bayesian prior elicitation on the efficacy of medical therapies in perianal fistulizing Crohn’s disease

**DOI:** 10.1093/ecco-jcc/jjag061

**Published:** 2026-05-10

**Authors:** Nurulamin M Noor, Haiyan Zheng, Zhi Cao, Gianmarco Caruso, Corey Voller, Rachel Cooney, Shahida Din, Hannah Gordon, Klaartje B Kok, James O Lindsay, Gordon W Moran, Kamal V Patel, Shaji Sebastian, Tim Raine, Sreedhar Subramanian, Ailsa L Hart, David S Robertson, Miles Parkes

**Affiliations:** Department of Gastroenterology, Cambridge University Hospitals NHS Foundation Trust, Cambridge, United Kingdom; Department of Medicine, University of Cambridge School of Clinical Medicine, Cambridge, United Kingdom; Department of Mathematical Sciences, University of Bath, Bath, United Kingdom; Medical Research Council Biostatistics Unit, University of Cambridge, Cambridge, United Kingdom; Medical Research Council Biostatistics Unit, University of Cambridge, Cambridge, United Kingdom; Department of Mathematical Sciences, University of Bath, Bath, United Kingdom; GI Unit, University Hospitals Birmingham NHS Foundation Trust, Birmingham, United Kingdom; Edinburgh IBD Unit, Western General Hospital, Edinburgh, United Kingdom; Translational Gastroenterology and Liver Unit, University of Oxford, Oxford, United Kingdom; Department of Gastroenterology, Royal London Hospital, Barts Health NHS Trust, London, United Kingdom; Department of Gastroenterology, Royal London Hospital, Barts Health NHS Trust, London, United Kingdom; Blizard Institute, Barts and The London Faculty of Medicine and Dentistry, Queen Mary University of London, London, United Kingdom; National Institute of Health Research Nottingham Biomedical Research Centre, University of Nottingham and Nottingham University Hospitals, Nottingham, United Kingdom; Department of Gastroenterology, St George’s University Hospitals NHS Foundation Trust, London, United Kingdom; IBD Unit, Department of Gastroenterology, Hull University Teaching Hospitals NHS Trust, Hull, United Kingdom; Department of Gastroenterology, Cambridge University Hospitals NHS Foundation Trust, Cambridge, United Kingdom; Department of Gastroenterology, Cambridge University Hospitals NHS Foundation Trust, Cambridge, United Kingdom; IBD Unit, St Mark’s Hospital, Harrow, United Kingdom; Medical Research Council Biostatistics Unit, University of Cambridge, Cambridge, United Kingdom; Department of Gastroenterology, Cambridge University Hospitals NHS Foundation Trust, Cambridge, United Kingdom; Department of Medicine, University of Cambridge School of Clinical Medicine, Cambridge, United Kingdom

**Keywords:** perianal fistulizing Crohn’s disease, PFCD, clinical trials, Bayesian statistics, prior elicitation

## Abstract

**Background & Aims:**

Robust evidence for most licensed Crohn’s disease therapies is lacking for perianal fistula outcomes due to a lack of dedicated clinical trials. This study aimed to use a Bayesian framework to determine the efficacy of medical therapies for perianal fistulizing Crohn’s disease (PFCD).

**Methods:**

A formal prior elicitation exercise was conducted by a group of 11 gastroenterologists and 5 statisticians. Consensus priors were developed leveraging both existing published data and clinical expertise, to determine one-year fistula remission rates for medical treatments with 5 different mechanisms of action (anti-TNF, anti-integrin, anti-IL-12/23, anti-IL-23, and JAK inhibitor). Consensus priors on efficacy of each treatment were determined relative to an elicited consensus prior for placebo control.

**Results:**

Consensus priors were obtained for the likelihood of fistula remission at 1 year. The prior mean, together with a 90% prior credible interval, of the one-year fistula remission rate was 0.22 (0.05, 0.46) for placebo, 0.58 (0.09, 0.96) for intravenous infliximab, 0.39 (0.06, 0.82) for adalimumab, 0.53 (0.09, 0.93) for subcutaneous infliximab, 0.24 (0.03, 0.60) for intravenous vedolizumab, 0.44 (0.05, 0.90) for upadacitinib, 0.34 (0.04, 0.77) for ustekinumab, and 0.36 (0.04, 0.82) for anti-IL-23 specific agents. Oral upadacitinib and subcutaneous infliximab demonstrated the highest probability for efficacy, alongside intravenous infliximab.

**Conclusions:**

We have conducted the first Bayesian prior elicitation exercise in inflammatory bowel disease. The generated priors could be used to enhance the design and analysis of clinical trials in PFCD by improving estimation of treatment efficacy, minimizing sample sizes, and potentially reducing the need for placebo control arms.

## 1. Introduction

Perianal fistulizing involvement occurs in approximately 25% of patients living with Crohn’s disease (CD),^1^^,^^2^ and is consistently rated as one of the most severe and treatment-refractory phenotypes, with a major impact on quality of life.^3^ Often resulting in high healthcare costs and healthcare resource utilization.^4^ Although there has been increasing focus on the biology underlying perianal fistulizing Crohn’s disease (PFCD); the etiology has remained poorly understood.^5^ While in luminal CD there have been major strides forward to improve outcomes for patients,^6^ outcomes and management of PFCD have remained largely unchanged over the last decade.^7^ Moreover, management of PFCD has been recognized to be sub-optimal, even at specialist, referral centres.^8^ As a result, this phenotype has been identified as one of the greatest areas of unmet clinical research need in inflammatory bowel disease (IBD), as part of a priority setting research partnership exercise conducted through the James Lind Alliance.^9^

A major issue that has hampered progress has been the lack of dedicated clinical trials assessing the efficacy of available therapies in PFCD.^10^ Most available data has been derived from exploratory *post-hoc* sub-group analyses of phase 3 or 4 clinical trials for luminal CD,^11^ resulting in a paucity of robust evidence, and high uncertainty around efficacy of treatments.^12^ To date, there has only been one adequately powered and dedicated randomized controlled trial (RCT) of a medical therapy used for registrational purposes in PFCD—that being the use of infliximab in the ACCENT II trial.^13^ However, conduct of dedicated clinical trials in PFCD pose additional challenges,^10^ including debate on appropriate endpoint selection and lack of validated outcome measures. In addition, unlike trials in luminal CD, where it may be possible to recruit relatively large patient cohorts, in PFCD, there is much greater difficulty with recruitment and meeting conventional power requirements. Therefore, any statistical approaches able to provide useful information from trials of small cohorts have great promise in the area of PFCD. Moreover, the potential risk of harm from use of placebo-controlled trials may be more acutely felt in this phenotype, with greater likelihood of progression to complications without appropriate disease control.^14^^,^^15^ Accordingly, it has become clear that alternative approaches are needed in clinical trials for PFCD.^16^ One potential solution to some of these challenges is to use Bayesian statistical approaches, which incorporate evidence from historical studies, including data synthesized from meta-analyses, together with knowledge from clinical experts.^17^^,^^18^

Classically, “frequentist” approaches to clinical trials have been used, which are based on the concept of repeated sampling and considering the frequency for events of interest (eg, rejection of the null hypothesis) over a defined period of time.^19^ These results have then been used to determine whether a treatment is efficacious or not compared to a control group.^20^ However, with the availability of data prior to initiation of a clinical trial including: high levels of clinical experience and expertise in both luminal and perianal indications, growing appreciation of key pathways underlying disease biology, accumulation of data over time (from multiple different sources), and uncertainty about interventions between clinicians, there has been recognition and appreciation for the potential of Bayesian methods to improve design and analysis of clinical trials.^21^ Despite widespread acknowledgement of the potential benefits of using Bayesian statistics, such approaches have rarely been used in IBD.

The uptake of Bayesian methods across clinical trials has been mostly restricted by a lack of expertise in how to utilize Bayesian statistics.^22^^,^^23^ A crucial first step to enable Bayesian analysis of any clinical trial is to establish a “prior distribution,” which captures relevant preliminary information about the quantity of interest (eg, the magnitude of clinical benefit with a treatment). When relevant historical data are not available or highly heterogeneous, prior distributions can be determined from a combination of published data and expert opinion. A formal process by which “priors” can be established is through an exercise that is typically referred to as elicitation. There have now been well-established examples of elicitation exercises being conducted to construct prior distributions that can inform subsequent clinical trials using a Bayesian framework, including for other immune-mediated-inflammatory diseases (IMIDs).^24^^,^^25^ These priors then being able to support the design of dedicated clinical trials through better prioritization and selection of treatments, more accurate sample size estimation, and potentially avoiding or reducing the need for placebo if an appropriate prior can be established for placebo control.

We sought to conduct an elicitation exercise to establish priors for the efficacy of licensed medical treatments and placebo control in PFCD. We report the methodology and the results of this exercise, to help stimulate further research and support dedicated clinical trials in this area of unmet clinical need.

## 2. Methods

### Pre-workshop meetings

In the months leading up to the elicitation workshop, a series of pre-workshop meetings were held by the steering committee comprising 5 clinical experts and 2 statisticians. A key goal of these meetings was to decide on the appropriate endpoint and medical therapies of interest for prior elicitation. After consideration of the available data in prior published studies, fistula remission at one year was chosen as the most relevant endpoint of interest for this elicitation exercise. With a myriad of scoring indices published for PFCD,^26^ the relative merits of quality of life, radiological, and clinical criteria to define fistula remission were considered. Clinical criteria to define fistula remission were selected to align with the greatest body of published data and recent meta-analysis of clinical outcomes across advanced therapies using this as the outcome measure of choice. Selection of fistula remission based on clinical criteria was also considered be consistent with recent guidance on important clinical trial outcomes for patients with PFCD.^27^

We sought to conduct prior elicitation for all licensed medical therapies in the context of CD. In the United Kingdom (United Kingdom), at the time of this workshop in 2024, there were 5 licensed classes of medication defined by mechanism of action (anti-tumor necrosis factor [TNF], anti-integrin, anti-interleukin [IL] 12/23, anti-IL23, Janus kinase [JAK] inhibitor). The following 7 licensed therapeutic options were selected for development of priors in this elicitation workshop:

- IV (Intravenous) infliximab (anti-TNF).
- SC (Subcutaneous) infliximab (anti-TNF).
- Adalimumab (anti-TNF).
- IV vedolizumab (anti-integrin).
- Ustekinumab (anti-IL12/23 p40 agent).
- Upadacitinib (JAK inhibitor).
- Anti-IL-23 specific p19 agents.

Given the greatest experience and expertise was with IV infliximab, it was agreed that this would likely be the intervention with the greatest certainty among individuals for their “priors.” Accordingly, the “prior” for IV infliximab was developed first and then for all the remaining intervention options, as well as placebo. At the time of the workshop, there was only one anti-IL-23 specific p19 agent licensed for use in Crohn’s disease in the United Kingdom (risankizumab). However, the consensus group was acutely aware of two further anti-IL-23 specific p19 agents due to be licensed for CD in the near future and where there would potentially be interest in developing a “prior” to take forward for potential use in a clinical trial setting. Notably, the expert group supported leveraging results from published *post-hoc* analyses and clinical experience from some anti-IL-23 agents for which clinicians already had fairly substantial experience in CD, to generate useful data for more recently available anti-IL-23 agents, for which there might have only been more limited experience or data. Therefore, the decision was taken to establish a prior across anti-IL-23 specific p19 agents.

In parallel, the statistical model underlying the prior elicitation was developed, extending the method proposed by Hampson (2014) for non-inferiority trials to superiority trials,^28^ modelling details provided (Supplementary Appendix). Following this, a structured questionnaire was developed (Table S1) for each clinical expert to complete. An R Shiny web application was developed to implement the statistical model established for deriving prior probability densities of the remission rates and the treatment effect, which describe the expert opinion over a range of plausible values implied by the responses to the questionnaire. This web application can further visualize those prior distributions, as well as allowing these priors to be altered in real time during the course of communication between a clinical expert and a statistician. As part of the development of the questionnaire and R Shiny application, a mock prior elicitation was held with 5 of the clinical experts, and their feedback incorporated. This focused on greater clarity in defining the outcome measure, wording of questions to establish certainty of effect for both placebo and active treatments, and plausible values for remission rates for placebo and active treatments. Crucially, clinicians were asked to estimate priors for maximal efficacy of each medical therapy, on the assumption that standard of care treatment was otherwise optimized. In addition to the above, a training video was developed by the clinical and statistical leads for this project to be watched by all invited experts. After confirmation of completing training video, each invited expert was provided a link to the R Shiny web app to view the platform that would be used to develop priors for the workshop. The final questions and linked script are provided (Table S1 and Figure S1, see online supplementary material for a color version of this figure).

Based on experience of the statistical team, it was determined that the elicitation process would be most optimal to perform during a face-to-face workshop. Prior work from the Sheffield Elicitation Framework had provided suggestion on the number of experts for prior elicitation (approximately 5–8),^29^^,^^30^ and this was considered in the context of other similar applications in immune-mediated diseases, which had reported group sizes of 12–15.^24^^,^^31^ Therefore, initially 12 expert UK clinicians were approached regarding their interest in taking part in this face-to-face prior elicitation workshop. Eleven of these individuals confirmed their availability to attend. These experts were chosen based on their experience relating to medical management of PFCD, including contribution to relevant national and international projects and guidelines. All 11 experts were sent the structured questionnaire to complete in advance of the workshop, along with access to a (simplified version of) the R Shiny application and a short training video. The responses to the questionnaire were analyzed by a wider team of statisticians in advance of the workshop (H.Z., Z.C., G.C., C.V., and D.S.R.).

### Statistical model

We followed the statistical methodology described in Hampson et al.,^28^ and applied in Hampson et al.^31^ We focused on the prior distributions for the probability of fistula remission at one-year on placebo and for each of the 7 therapy options. Full details of statistical model are provided (Supplementary Appendix).

### Elicitation workshop

The workshop took place as a face-to-face meeting at the Wellcome Collection in London, United Kingdom on November 11, 2024 (Table S2). The first part of the day focused on describing the aims of the workshop and reviewing current medical treatments and endpoints in PFCD. In addition, further training on Bayesian statistics for clinical research was provided, and the overall approach to prior elicitation was discussed.

The second part of the day focused on the formal prior elicitation process. The initial opinions of the individual clinical experts had already been established via the structured questionnaire that each had completed in advance of the elicitation workshop. At the workshop itself, each expert then had a one-to-one meeting with one of the statisticians present, during which the answers provided were adjusted as required to ensure that the prior distributions adequately reflected the experts’ opinions for each therapy, using the R Shiny web application for visualization. To reduce the possibility of a systematic learning effect in the prior elicitation, the discussion with each expert started with IV infliximab (as this was thought to be the therapy option with the most certainty around treatment effect) and then the other 6 therapies were randomized in order. To reduce the risk of experts being unduly influenced by others, questionnaire-related discussion of priors before the one-to-one meetings was not allowed to take place.

After all experts had completed their one-to-one meetings, the individual prior distributions for each therapy were presented to all experts as the basis for a group discussion of the opinions. Individuals could adjust their prior based on this group discussion before having their individual results “locked.” Consensus prior distributions were then developed via behavioral aggregation (collective discussion by the expert group on a topic rather than trying to establish a single unified “correct” answer) following the Sheffield Elicitation Framework.^29^^,^^30^ Moreover, there was no weighting of priors in this elicitation exercise, and all participating experts were considered equally “expert” for this process. These discussions were facilitated by 2 clinicians (N.M.N. and M.P.) and 2 statisticians (H.Z. and D.S.R.). Discussions included clinical aspects such as what control treatment experts had in mind when eliciting the relative treatment benefit. Visualizations of the consensus prior were created for each therapy and presented to the group (along with their interpretation) to aid discussion.

### Analysis and aggregation of beliefs and uncertainties

Opinion on plausible values of p_0_, as well as the associated level of uncertainty about such values, was uniquely defined by two parameters of a beta distribution. Two structured questions (Table S2; Q1 and Q2) were designed to ascertain those parameters. Beta prior density curves for p_0_ were then visualized collectively and presented to all experts. This was followed by reflection on individual density curves and further fine tuning. Consensus was reached by vote to a pair of answers to Q1 and Q2 that best represent the collective opinion on values of p_0_. Opinion on the relative efficacy of each therapy option against placebo control (θ_j_) was modeled as a normal distribution. Plausible values of θ_j_ and the level of uncertainty were determined by the normal distribution’s mean and standard deviation, as implied by the experts’ answers to two other questions (Table S2; Q3 and Q4).

For individual “prior” elicitation, priors for placebo and IV infliximab were developed first and then for all the remaining intervention options. Given the greatest experience with IV infliximab and placebo, it was agreed that these would likely be the interventions with the greatest certainty among individuals for developing their “priors.” To minimize the effect of specific sequences, a randomized approach was taken for all subsequent interventions. The ordering of elicitation for each of the interventions after placebo and IV infliximab was randomized for each individual expert. This simple randomization was performed using random permutations of the therapies for each clinical expert. After all experts had completed their one-to-one meetings, the individual prior distributions at this stage were “locked” for each therapy and were presented to all experts as the basis for a group discussion to allow an overall consensus prior to be established.

The group reconvened to share and discuss their answers alongside the visualization of the individual density curves for p_0_ and p_j_. Experts took this opportunity to communicate the scenarios in mind for elicitation and eventually reached a consensus. The elicitation process was iterative. The established statistical model and the bespoke R shiny web application allowed quick visualization of the prior density curves, which facilitated the elicitation of opinion for 7 therapy options on the same day. A summary of the entire prior elicitation process is shown in Figure S2 (see online supplementary material for a color version of this figure).

## 3. Results

Eleven experts in CD attended the Bayesian prior elicitation workshop. Individual answers by clinical experts were compiled in response to a structured questionnaire covering efficacy of 7 medical therapy options for PFCD, with each treatment in comparison to placebo control.

Clinical experts had their priors for each treatment reviewed before the workshop and after review of the contemporary evidence for each treatment at the workshop. Some experts changed their initial answers for reasons including: (1) misinterpretation of some questions, (2) a deeper understanding developed about probability densities so experts were able to fine-tune their opinion expressed for certain quantities, (3) greater awareness and alignment of the contemporary evidence-based after review of the published data for each medical therapy and after the initial group discussion. Individual priors for each medical therapy were established following individual one-to-one review, discussion with statisticians and then following group discussion an overall consensus prior was established for each medical treatment.

**Figure 1.**
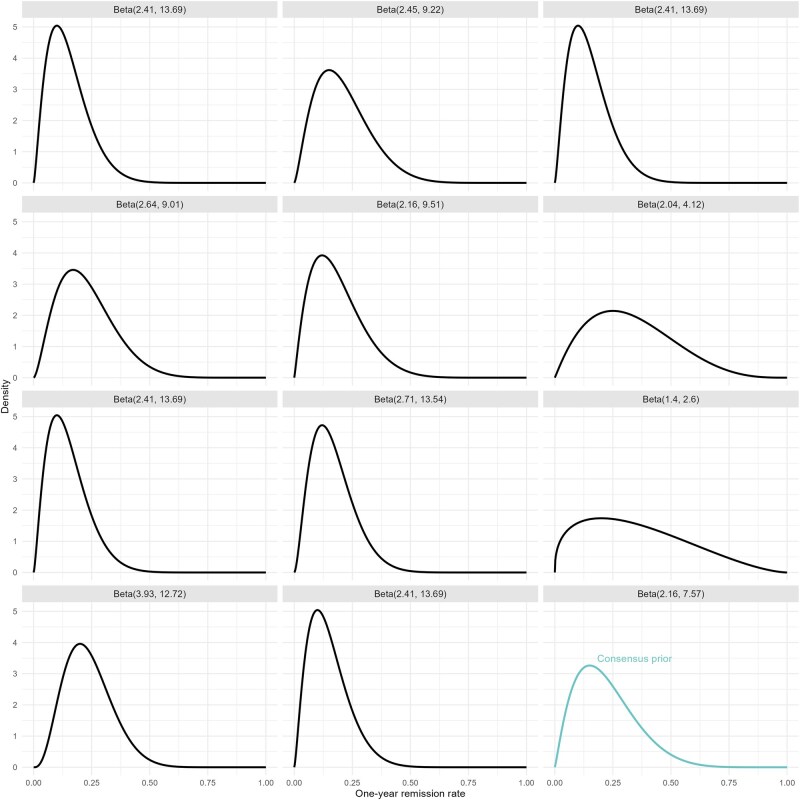
Individual prior distributions and a consensus prior for 1-year fistula remission rate on placebo.

Individual prior distributions for the one-year fistula remission rate on placebo were developed (Figure 1), with a consensus for the contemporary value being most likely lower than 20%. The resulting consensus prior had a mean of 0.22, with probability of 90% that p_0_ lies between 0.05 and 0.46. This consensus prior was then fixed for eliciting the treatment-effect for each of the 7 therapeutic options.

**Figure 2.**
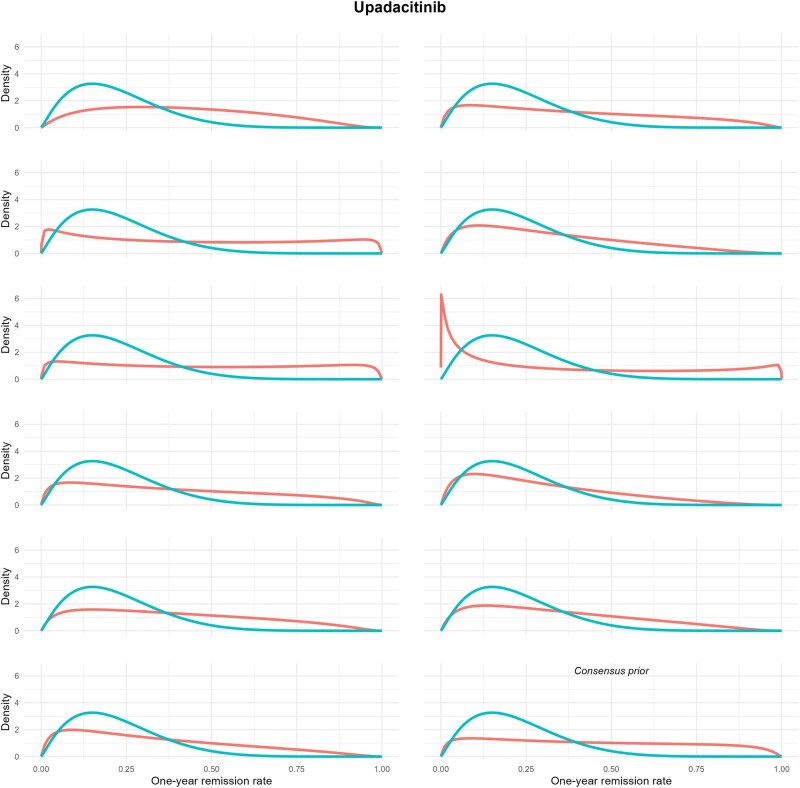
Individual prior distributions and consensus prior for 1-year fistula remission rate on upadacitinib, in contrast to the consensus prior distribution on placebo.

Using the example of upadacitinib, individual prior distributions for the one-year fistula remission rate on upadacitinib were established in comparison to placebo (Figure 2). These sets of opinion were diverse, suggesting various ranges of plausible values among the clinicians. While there was high level of certainty for the placebo elicited prior for p_0_ that fell within (0.05, 0.30), there were higher levels of uncertainty for the probable values of treatment effect for upadacitinib. This exercise was undertaken for all the remaining 6 therapeutic options, demonstrating varying strength of priors for each treatment compared to placebo, with a complete visualization of individual prior distributions presented (Figures S3-S8, see online supplementary material for a color version of these figures).

**Figure 3.**
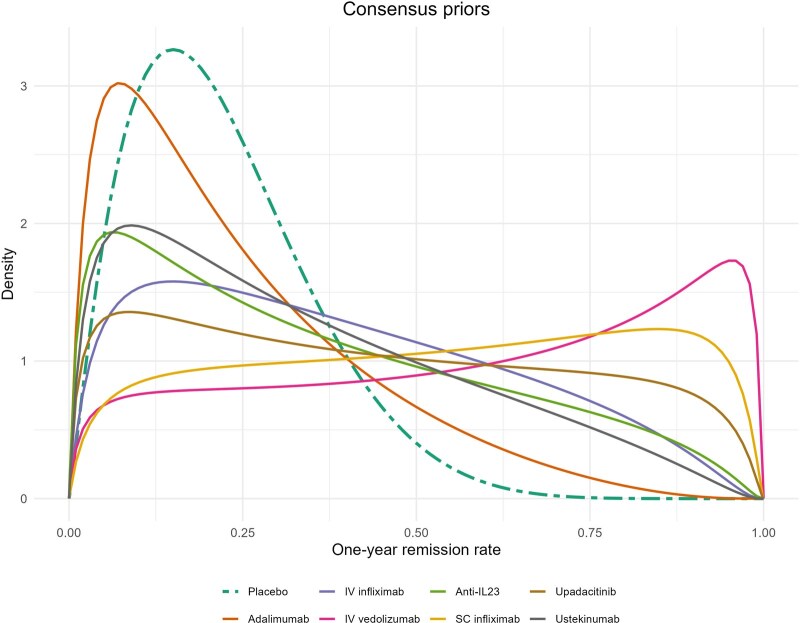
Consensus prior for the 1-year remission rates on placebo and medical therapies.

Consensus priors were obtained after the group discussion. As shown in Figure 3, the prior mean, together with a 90% prior credible interval, of the one-year fistula remission rate was 0.22 (0.05, 0.46) for placebo, 0.39 (0.06, 0.82) for adalimumab, 0.36 (0.04, 0.82) for anti-IL-23 specific agents, 0.44 (0.05, 0.90) for upadacitinib, 0.34 (0.04, 0.77) for ustekinumab, 0.58 (0.09, 0.96) for IV infliximab, 0.53 (0.09, 0.93) for SC infliximab, and 0.24 (0.03, 0.60) for IV vedolizumab.

**Figure 4.**
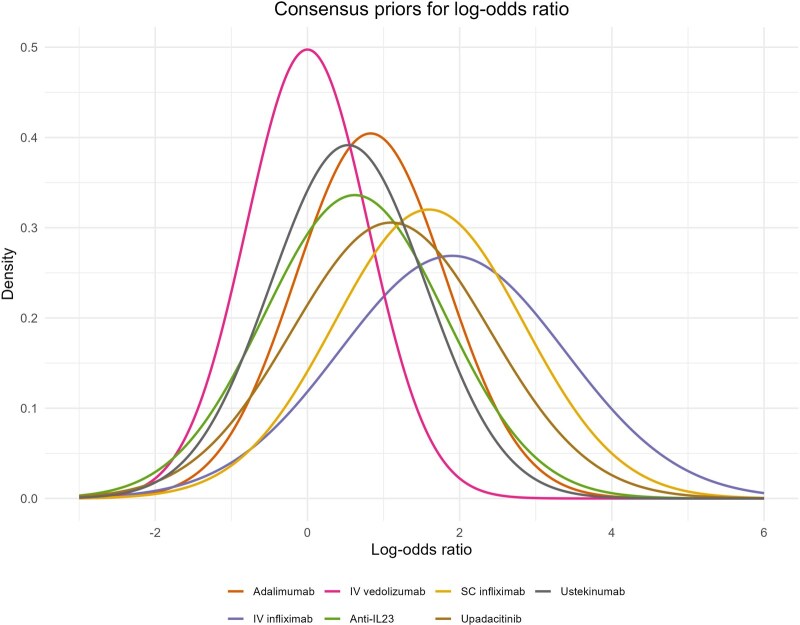
Consensus prior for the log-odds ratio of the 1-year remission rate of 7 medical therapy options compared with placebo.

Consensus priors for the log-odds ratio were also obtained for each of the 7 medical therapy options, demonstrating a potential hierarchy of likely efficacy for each of the licensed therapeutics (Figure 4). Priors elicited for the treatment effect on both the log-odds ratio scale and for one-year fistula remission rates, are summarized in Table 1.

**Table 1.** Quantitative summary of consensus priors for the 1-year remission rates on placebo and medical therapies, including for the log-odds ratio.

	Prior mean (90% credible interval) for p_0_	Prior distribution
**Placebo**	0.22 (0.05, 0.46)	Beta (2.16, 7.57)
	**Prior mean (90% credible interval) for p_j_**	**Prior distribution for θ_j_**
**IV infliximab**	0.58 (0.09, 0.96)	N (1.90, 2.20^2^)
**Adalimumab**	0.39 (0.06, 0.82)	N (0.83, 0.97^2^)
**SC infliximab**	0.53 (0.09, 0.93)	N (1.60, 1.55^2^)
**IV vedolizumab**	0.24 (0.03, 0.60)	N (0, 0.64^2^)
**Upadacitinib**	0.44 (0.05, 0.90)	N (1.10, 1.70^2^)
**Ustekinumab**	0.34 (0.04, 0.77)	N (0.53, 1.04^2^)
**Anti-IL-23**	0.36 (0.04, 0.82)	N (0.62, 1.41^2^)

Exploring the example of upadacitinib in more detail (Figure 2), the elicited prior for the efficacy of upadacitinib was N(1.10, 1.70^2). Breaking down this result, on average remission was expected to be about 3 (explicitly, exp(1.10)≈3) times more likely to be achieved for patients receiving upadacitinib than placebo. However, the associated uncertainty with this remission rate for upadacitinib was high. Specifically, 68% of the time, the value of the log-odds ratio fell within 1 (2) standard deviations of the mean, ie, between −0.6 and 2.8 (−2.3 and 4.5). Putting this “prior” for upadacitinib into context, these figures demonstrate that the odds of remission with upadacitinib could be substantially greater than placebo control (explicitly, exp(4.5)≈90), but it is also plausible that the odds for remission with upadacitinib could be similar to placebo control (explicitly, exp(-2.3)≈0.1).

An important additional factor is how priors can be used to support Bayesian sample size calculations. In this regard, when using this N(1.10, 1.70^2) “prior” in the design or analysis of a new comparative clinical trial of upadacitinib, such information would be equivalent to a prior effective sample size (ESS) of (σ/1.70)^2. The prior ESS translates the information captured in the “prior” on the scale of a clinical trial sample size—therefore giving an indirect measure on the strength of the prior distribution. In a potential clinical trial, it may be expected that the standard error, σ, would likely take a value larger than 1.70, due to either a smaller trial sample size or high variability in the response. For illustration purposes, assuming σ = 3.5, the prior would have the same strength as if 5 patients were treated in a trial comparing upadacitinib versus placebo. Assuming a larger σ = 8, this prior would be equivalent to the data from 22 patients–helping to substantially lower the required sample size for any proposed clinical trial. Using the priors generated from this study, effective sample size reduction for any potential trial is demonstrated (Figure S9, see online supplementary material for a color version of this figure). A formal Bayesian sample size estimation using the priors elicited goes beyond the scope of this paper, as many additional considerations need to be accommodated depending on the clinical and statistical context. We refer the interested readers to further articles encompassing a range of methodology on sample size determination, covering both hybrid frequentist-Bayesian approaches and fully Bayesian approaches.^32–34^

As well as helping determine sample size and design potential clinical trials as “design priors,” the elicited prior could also be used in the analysis of a new trial as “analysis priors.” If a new comparative clinical trial were to enroll 80 patients, randomly assigned to receive upadacitinib or placebo by a 1:1 ratio, the prior would be updated to give a posterior distribution, N((0.381 + 20/σ^2*x̄)/(0.346 + 20/σ^2), 1/(0.346 + 20/σ^2)), where x̄ denotes the sample mean log-odds ratio and σ the known standard deviation.

## 4. Discussion

Our study is the first Bayesian prior elicitation exercise for PFCD. We have established formal priors for likely efficacy of placebo and 7 licensed medical therapies (covering 5 different mechanisms of action) in PFCD. These priors were leveraged from existing published data and clinical expertise. Notably priors are not “new” efficacy data themselves but rather estimates which can be used to support more robust and accurate trial design and analysis. Clinical criteria to define fistula remission were selected to align with the greatest body of published data, therefore our findings have high external validity and potential utility for conduct of pragmatic clinical trials.

The consensus priors, especially those for θ_j_, may be used to improve the design and add power to the analyses of future PFCD clinical trials. These priors may be particularly useful in the context of recent evidence about what might be considered a small, moderate or large beneficial effect of treatments in IBD.^35^ The prior elicitation exercise itself confirmed confidence in IV infliximab for cases of PFCD, being consistent with published clinical trial data,^13^^,^^36^ and global clinical practice.^7^ Importantly, despite an absence of clinical trial data, similarly high priors for likely efficacy were observed for both SC infliximab and oral upadacitinib, although there was less certainty around these priors. These data suggest that subcutaneous infliximab and oral upadacitinib should perhaps be prioritized for evaluation in clinical trials. Indeed, the high priors for upadacitinib would be in keeping with data to support the importance of the JAK-signal transducer and activator of transcription (JAK-STAT) pathway in pathophysiology of PFCD.^37^ Moreover, these data would also be consistent with emerging clinical data to support the promising role of upadacitinib for the treatment of PFCD via inhibition of this JAK-STAT pathway.^11^^,^^38^

At the time of the workshop, there was only one anti-IL-23 specific p19 agents licensed for use in Crohn’s disease in the UK (risankizumab). However, the consensus group were acutely aware of two further anti-IL-23 specific p19 agents due to be licensed for CD in the near future (mirikizumab and guselkumab). Therefore, a consensus prior was established across anti-IL-23 specific p19 agents. This is particularly noteworthy given that there is a dedicated placebo-controlled PFCD trial that has completed recruitment and awaiting reporting for use of guselkumab (FUZION trial; NCT05347095). The findings demonstrated from the FUZION trial will be helpful to compare with the prior established for anti-IL-23 specific p19 agents, and the results could help to inform future updates to this specific prior result as well.

Putting our results into context of the known literature, there have been several attempts to meta-analyze the effects of both placebo and medical treatments in PFCD. These have all highlighted the high heterogeneity between studies, with the majority of data obtained from exploratory *post-hoc* analyses of clinical trials in luminal CD.^12^^,39,^^40^ Reliance on *post-hoc* analyses is particularly problematic given that there is; no preservation of randomization between intervention arms, small numbers of included patients with PFCD, the trials themselves being designed for luminal CD outcomes, resulting in minimal and often sparse data collected on perianal outcomes. Accordingly, there has remained much uncertainty about the efficacy of medical treatments and placebo response/remission rates in PFCD.

With regards to application of Bayesian statistics to a clinical trial in IBD, this was first performed for the evaluation of secukinumab in CD.^41^ Although, secukinumab was not shown to be effective in CD, the use of Bayesian historical controls enabled investigators to get to this answer sooner, and with fewer patients randomized to placebo control than would otherwise have been the case. More recently, there was a single-arm triple combination therapy trial of vedolizumab, adalimumab, and methotrexate in luminal CD, the EXPLORER trial.^42^ In EXPLORER, priors were selected for each of the treatments as monotherapy and for placebo, based on aggregate-data meta-analyses. These priors were developed as part of a *post-hoc* Bayesian analysis and demonstrated that triple combination therapy might be more effective than any of the treatments as monotherapy, with high probability of efficacy over placebo. However, once again considering the pitfalls of *post-hoc* analyses, it has been widely accepted that future applications in CD should formally develop “priors” before any clinical trial in order to undertake *a priori* Bayesian analyses–helping to maximize robustness and confidence in trial results.^43^

Our study has several strengths. We have conducted the first formal Bayesian prior elicitation exercise for PFCD, and we believe that this is also the first such exercise in the field of IBD. Additionally, while previous Bayesian prior elicitation exercises in other IMIDs have sought to investigate one intervention against a control,^24^^,^^31^ we have developed formal consensus priors for 7 therapeutic interventions and placebo, from one prior elicitation workshop. A key strength of the prior elicitation process and from combining both published literature and expert consensus, was that heterogeneity between studies could be accounted for. Notably, standard of care alongside “placebo control” was taken into account, as clinicians were asked to estimate priors based on maximal likely efficacy of medical therapies, including factors such as antibiotic use, dietary optimization, and removal of seton sutures. One of the key strengths of these generated priors is that they have been able to go beyond *post-hoc* analyses from RCTs in relatively narrow populations, to also consider data from more broad “real-world” cohorts as well as leveraging the considerable expertise and experience of the prior elicitation group. Therefore, these findings should have potentially much greater utility for designing and analyzing clinical trials compared to other techniques such as using point estimates generated from meta-analyses of RCT sub-groups. These results have potentially important implications to inform the design and conduct of future clinical trials in PFCD,^44^ and support implementation of a potential Bayesian framework to speed up the efficiency of such trials.^45^ Notably, the prior established for placebo might allow reduction or perhaps even negate the need for placebo control arms in PFCD clinical trials. This work is especially pertinent, given recent guidance from the United States Food and Drug Administration encouraging use of Bayesian statistics to speed up the rate at which answers can be obtained from clinical trials.^46^

Our study also has limitations. First, given the high level of patient heterogeneity it has been difficult for PFCD trials to establish a single “standard of care.” For this study “optimal” standard of care was assumed for the generation of each medication “prior.” It is likely that other factors such as antibiotic use/duration, concomitant immunomodulator use/duration, dietary optimization, timing of seton removal and many other factors may also play a role. However, these additional factors, typically referred to as “intercurrent events” would also be present in any potential clinical trial of PFCD. After detailed consideration of different subtypes of disease, clinical consensus was reached to focus on developing “priors” for PFCD overall and not for each of the distinct classification subtypes of PFCD.^47^^,^^48^ Importantly, it was felt, that these findings could potentially be extended or validated in future to distinct fistula classification subtypes. Moreover, there has been an appropriate increasing focus in recent years on quality-of-life measures,^49^ and radiological measures of outcome in PFCD.^50^ When selecting the outcome measure for this prior elicitation exercise, we opted for the outcome measure most commonly reported for medical therapies in the field, supported by recent meta-analysis data and being consistent with recent guidance for suggested clinical trial outcomes in patients with PFCD.^27^ Therefore, it is anticipated that the findings from this study would have high external validity and applicability to clinical settings around the world. Given the relatively intense nature of the elicitation process, including workload both prior to and after the face-to-face workshop, this was conducted in the United Kingdom but with experts from multiple and diverse centers. Future work could seek to validate these findings internationally, including using composite measures and/or imaging-based evaluation using magnetic resonance imaging (MRI). We would encourage any potential future studies to report on group size and composition to support any future methodological work examining impact this may have on prior elicited findings. For the methodological process of this elicitation exercise, all clinicians established a prior for placebo and IV infliximab first, followed by all the remaining options—with randomization to different sequences for each individual. However, priors could have been established for other newer treatments first. It is possible that the ordering or sequence of treatments could have impacted on the findings. Although, the potential impact of elicitation sequence was mitigated by randomizing the ordering of prior elicitations for each individual expert.

It is well-recognized that medical treatment is just one aspect of PFCD management. There are many other crucial aspects which align with the principles of wound healing, including adequacy and timing of surgical management. Just as there is an unmet need for research of medical therapies, there is similarly a need to establish greater evidence around surgical interventions in PFCD. This study provides a template for any similar such prior elicitation exercise to be conducted to establish priors for “efficacy of surgical interventions” and to design dedicated surgical PFCD trials. Moreover, this exercise underscores the fact that currently licensed medical therapies may only offer partial benefit for patients, and that there remains a clear need for novel treatments and approaches in PFCD.^51^ Indeed, with the availability of new licensed therapeutics in CD or with novel data generated for currently licensed therapeutics, updating and/or generating new priors could help to maintain the utility of these estimates to inform design and analysis for PFCD clinical trials.

In summary, despite being a major area of unmet clinical need, there is a paucity of evidence for medical therapies in PFCD and a lack of dedicated clinical trials. The results from this study may better inform the design, conduct, and analysis of phenotype-specific PFCD trials. The “priors” we generated have multiple potential benefits, including prioritizing interventions for clinical evaluation, enabling the incorporation of relevant information from external sources, supporting sample size determination, and potentially reducing the need for placebo control in future dedicated PFCD clinical trials. These findings and the methodology used also have major potential to be applied across the field to support greater adoption of Bayesian clinical trials in IBD.

## Supplementary Material

jjag061_Supplementary_Data

## Data Availability

All data on generated priors have been provided in this publication. For any additional data queries regarding please contact the corresponding author.
