# Supplementary material for: Bayesian prior elicitation on the efficacy of medical therapies in perianal fistulizing Crohn’s disease
Source: J Crohns Colitis. 2026 May 10;20(5):jjag061. doi: 10.1093/ecco-jcc/jjag061 (PMC13157336; doi:10.1093/ecco-jcc/jjag061)
Supplement: jjag061_Supplementary_Data [file jjag061_supplementary_data.docx]

**Supplemental Appendix**

**Supplementary Methods**

Statistical model

We followed the statistical methodology described in Hampson et al. (2014),^20^ and applied in Hampson et al. (2015).^23^ We focused on the prior distributions for the probability of fistula remission at one-year on placebo and for each of the seven therapy options.

Denoting $p_{0}$ as the probability of remission at one-year on placebo, and denoting $p_{j}$ the probability of remission at one-year on each of the seven therapy options, labelled j = 1, …, 7. The log-odds ratio for efficacy for therapy j, was defined as:

$$\theta_{j}=log[\frac{p_{j}(1- p_{0})}{p_{0}(1- p_{j})}]$$

This was used to compare each therapy with the placebo control. Positive values of $\theta_{j}$ imply that therapy j is superior to placebo and conversely for negative values. The relative risk is another frequently used outcome in the literature, and the relationship between the two outcomes was communicated to the clinicians during the workshop.

Following Hampson et al. (2014),^24^ we elicited priors for $p_{0}$ and $\theta_{j}$. A beta distribution was used to model $p_{0}$ whilst a normal distribution was used to model $\theta_{j}$. The prior distribution for $p_{j}$ was then (numerically) derived from these two distributions. Although $\theta_{j}$ is defined as the log odds ratio, all 11 expert clinicians were asked questions on the probability difference scale for ease of convenience for the clinicians.

**Supplementary Table 1. Consensus answers to Q1-Q4 agreed by the group.**

|  | Q1 | Q2 | Q3 | Q4 |
| --- | --- | --- | --- | --- |
| Placebo | 0.15 | 0.30 | - | - |
| IV infliximab | - | - | 0.90 | 0.70 |
| adalimumab | - | - | 0.80 | 0.40 |
| SC infliximab | - | - | 0.90 | 0.65 |
| IV vedolizumab | - | - | 0.50 | 0.10 |
| upadacitinib | - | - | 0.80 | 0.50 |
| ustekinumab | - | - | 0.70 | 0.30 |
| Anti-IL-23 | - | - | 0.70 | 0.35 |

Q1: What do you think the one-year remission rate for patients with fistulising perianal Crohn's disease treated with placebo is?

Q2: Provide a proportion such that you are 75% sure that the true one-year remission rate on placebo does not exceed this value.

Q3: Assuming the patient tolerates the drugs, what is the chance that the one-year remission rate on IV infliximab is higher than that on placebo?

Q4: Assuming the patient tolerates the drug, what is the chance that the one-year remission rate on IV infliximab exceeds that on placebo by more than 20%?

**Supplementary Table 2. Timetable for the elicitation workshop.**

| Time allocation  (minutes) | Activity |
| --- | --- |
| 40 | Introduction to the project and aims, review of current medical treatments and endpoints in perianal Crohn’s disease |
| 90 | Training on Bayesian methods and approach to prior elicitation |
| 75 | Parallel one-to-one review and discussion of individual priors with statisticians |
| 75 | Presentation of individual priors and group discussion to reach consensus |
| 30 | Presentation of consensus priors and final discussion |

**Supplementary Figure 1. R Shiny web app worked example for prior elicitation.**

**
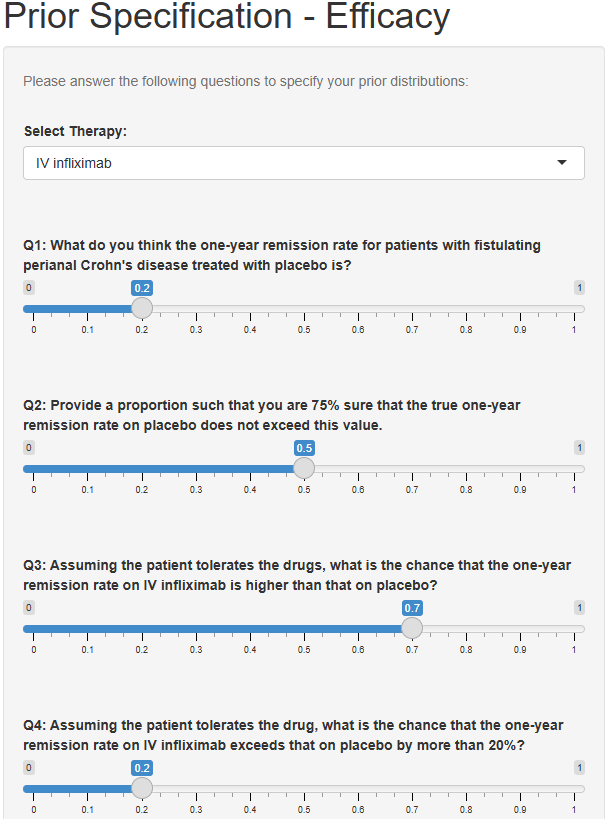
**

**Supplementary Figure 2. Overview of prior elicitation process.**

**
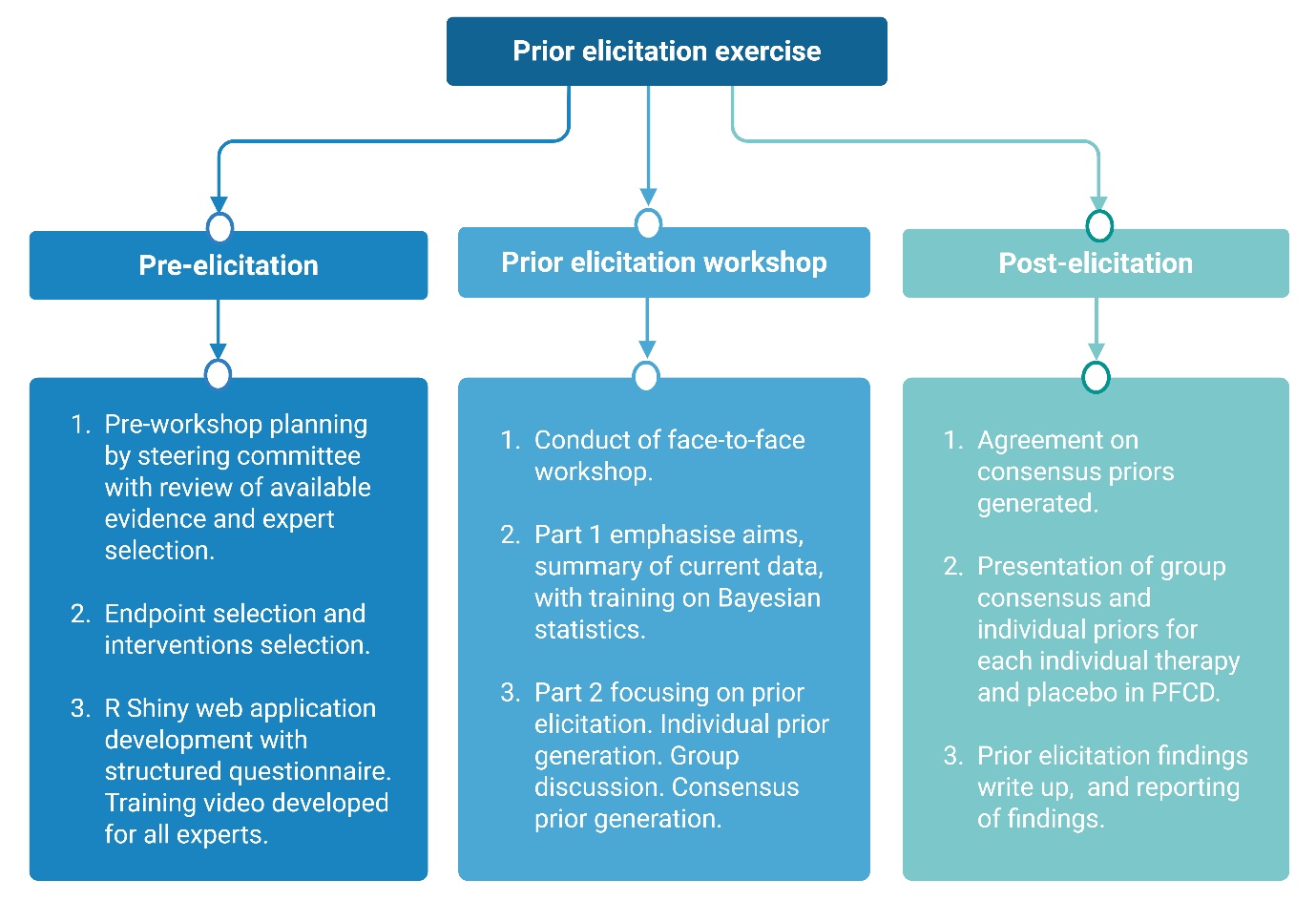
**

**Supplementary Figure 3. Individual prior distributions for the one-year remission rate on IV infliximab (with consensus prior for the one-year remission rate on placebo).**

**
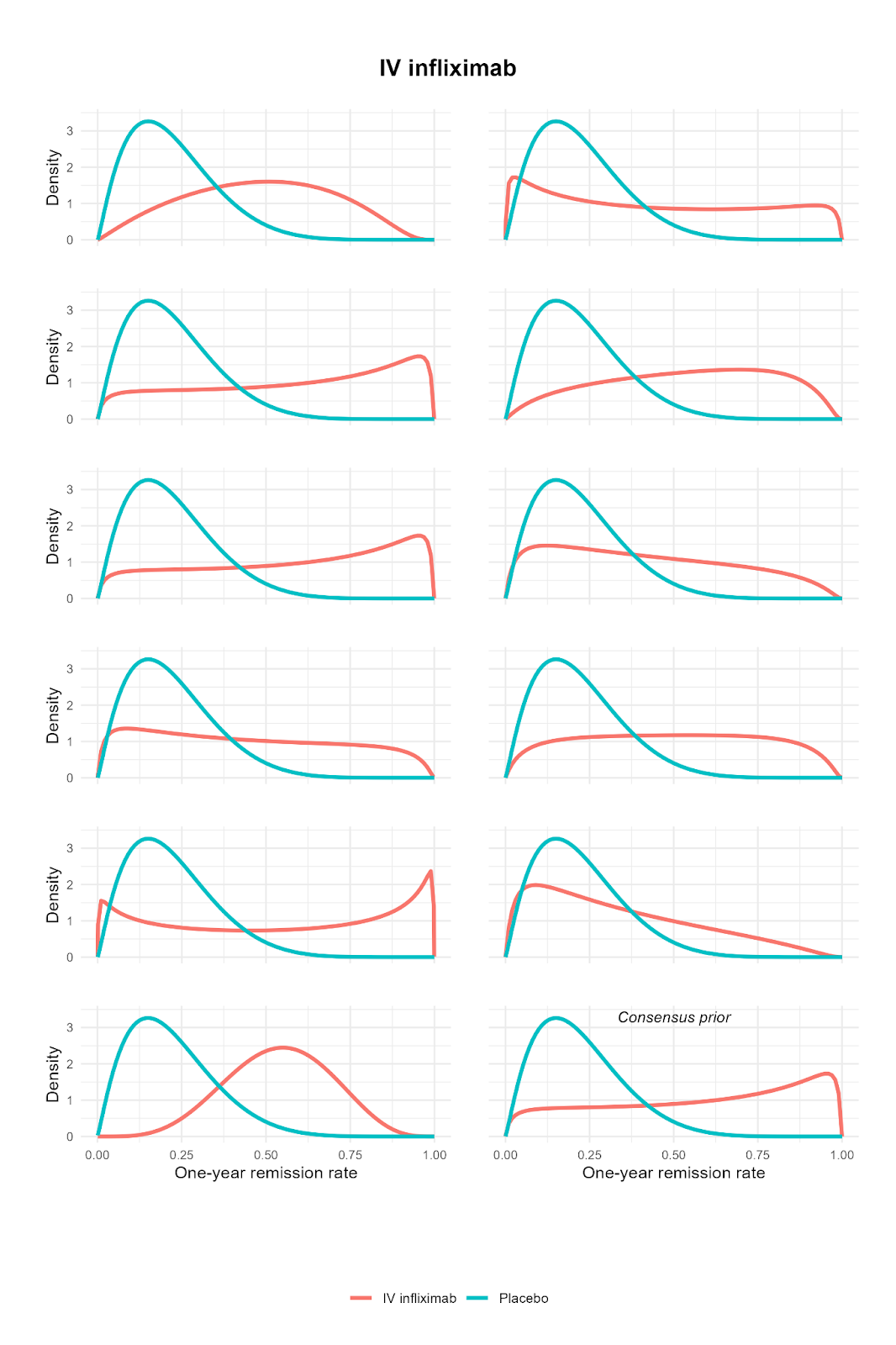
**

**Supplementary Figure 4. Individual prior distributions for the one-year remission rate on adalimumab (with consensus prior for the one-year remission rate on placebo).**

**
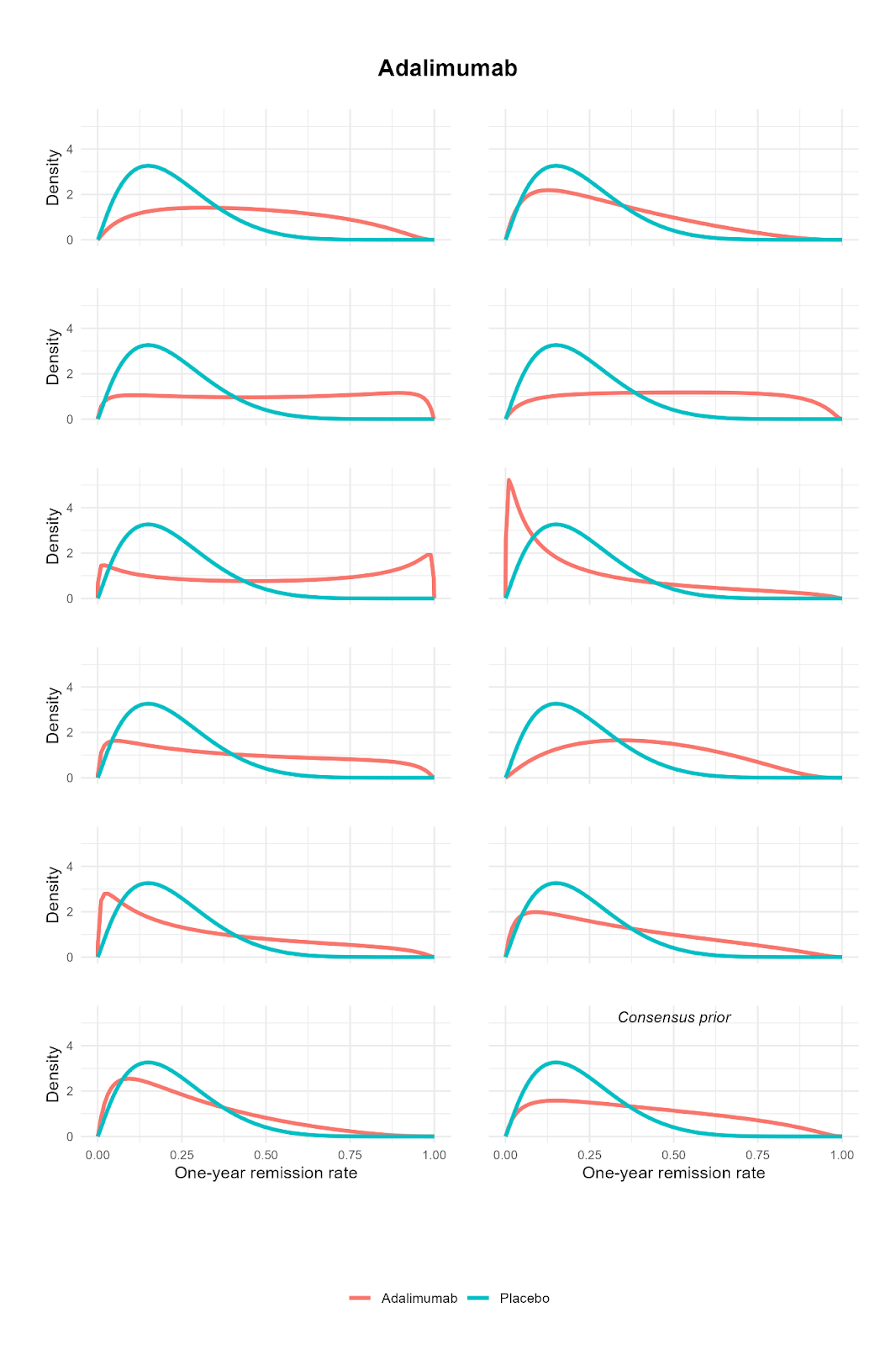
**

**Supplementary Figure 5. Individual prior distributions for the one-year remission rate on SC infliximab (with consensus prior for the one-year remission rate on placebo).**

**
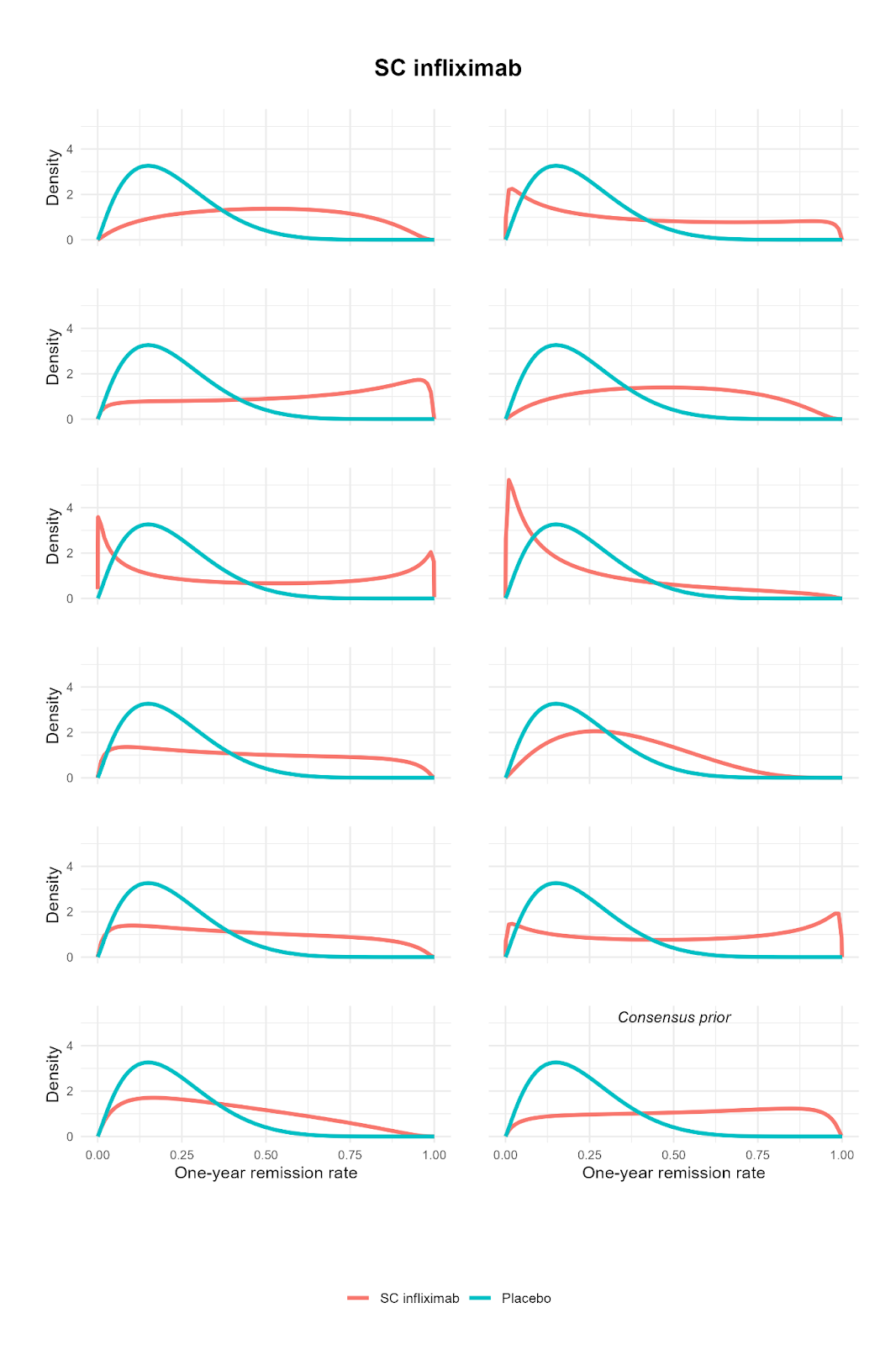
**

**Supplementary Figure 6. Individual prior distributions for the one-year remission rate on IV vedolizumab (with consensus prior for the one-year remission rate on placebo).**

**
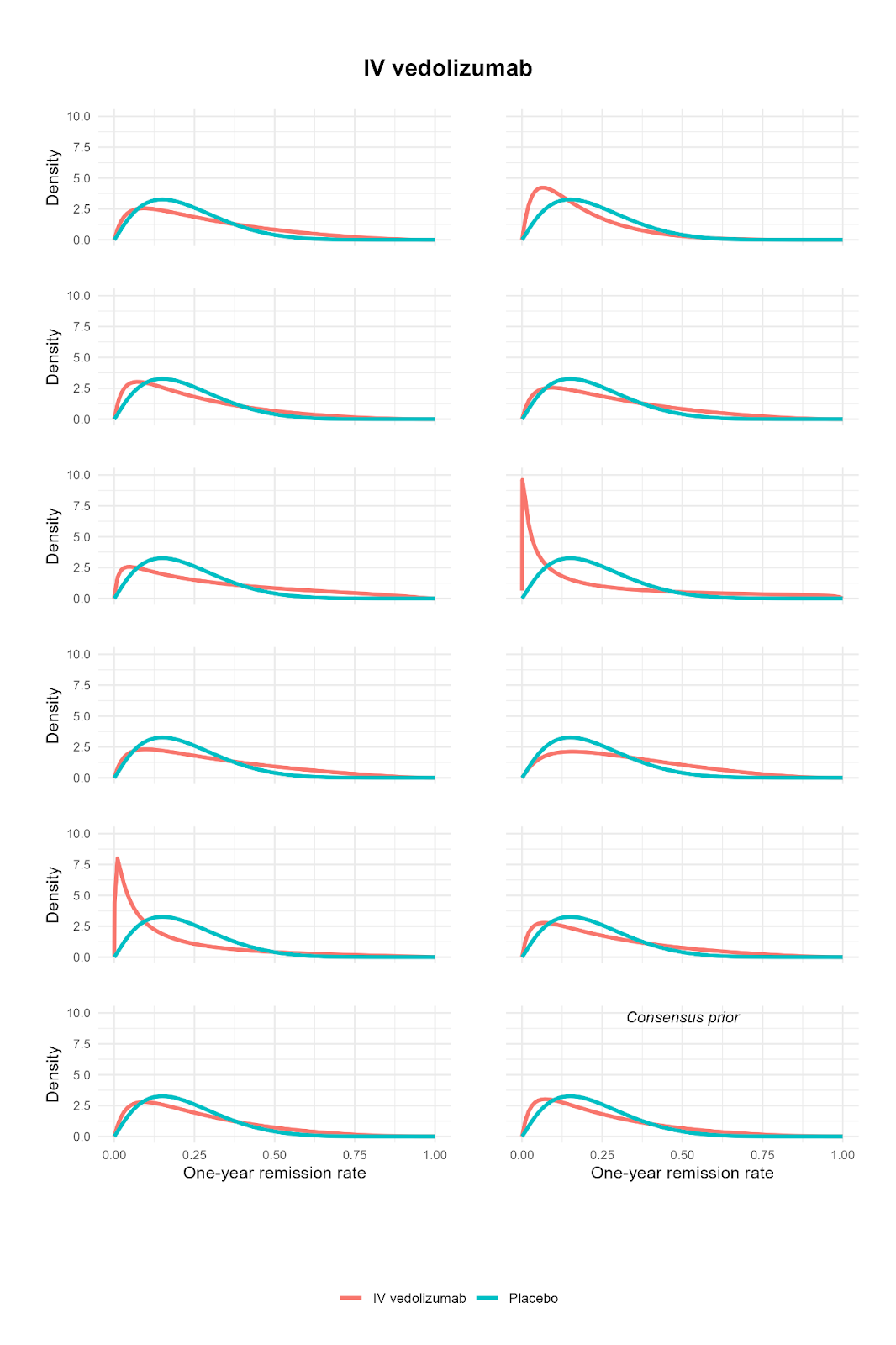
**

**Supplementary Figure 7. Individual prior distributions for the one-year remission rate on ustekinumab (with consensus prior for the one-year remission rate on placebo).**

**
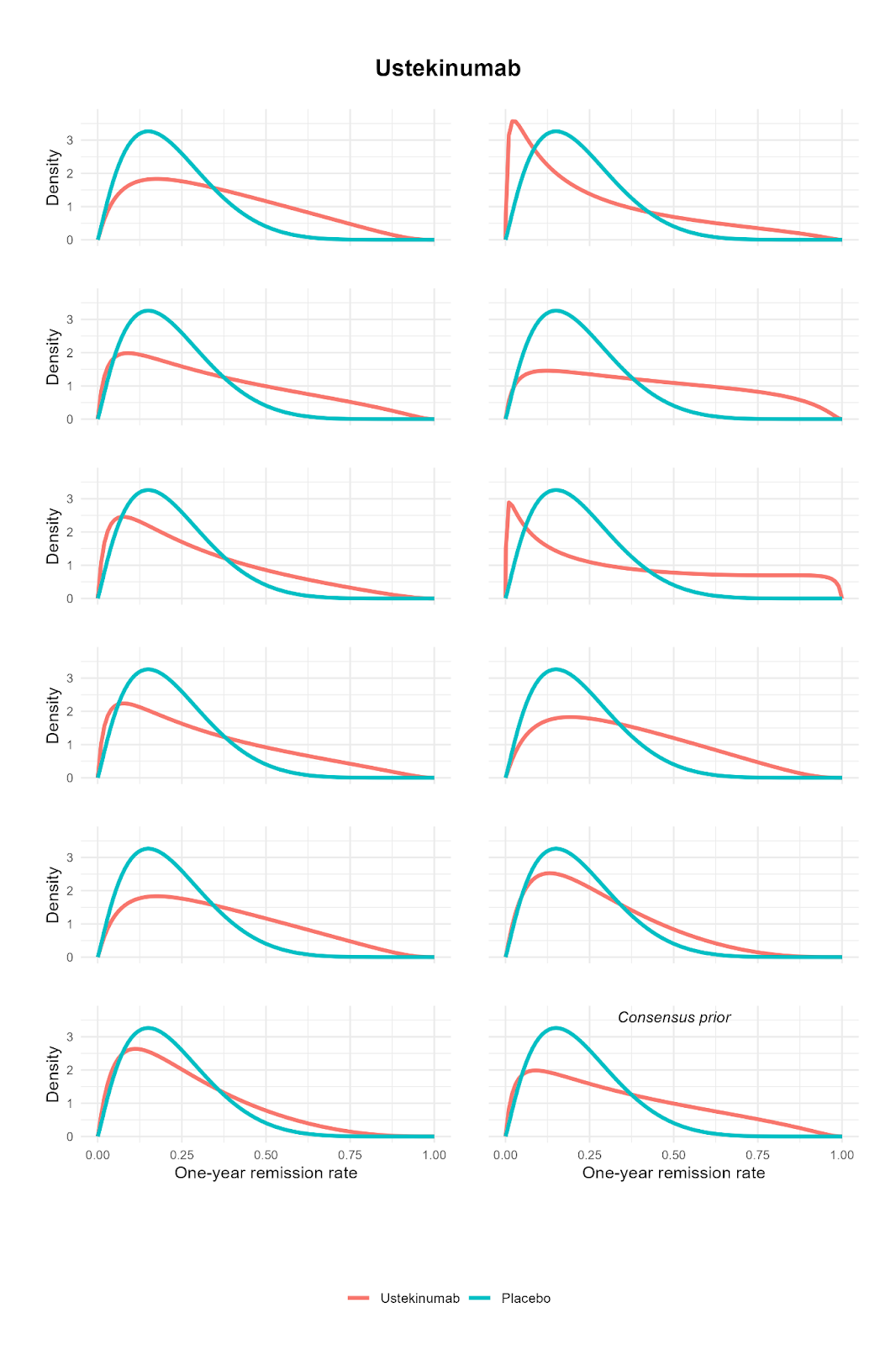
**

**Supplementary Figure 8. Individual prior distributions for the one-year remission rate on anti-IL-23 (with consensus prior for the one-year remission rate on placebo).**

**
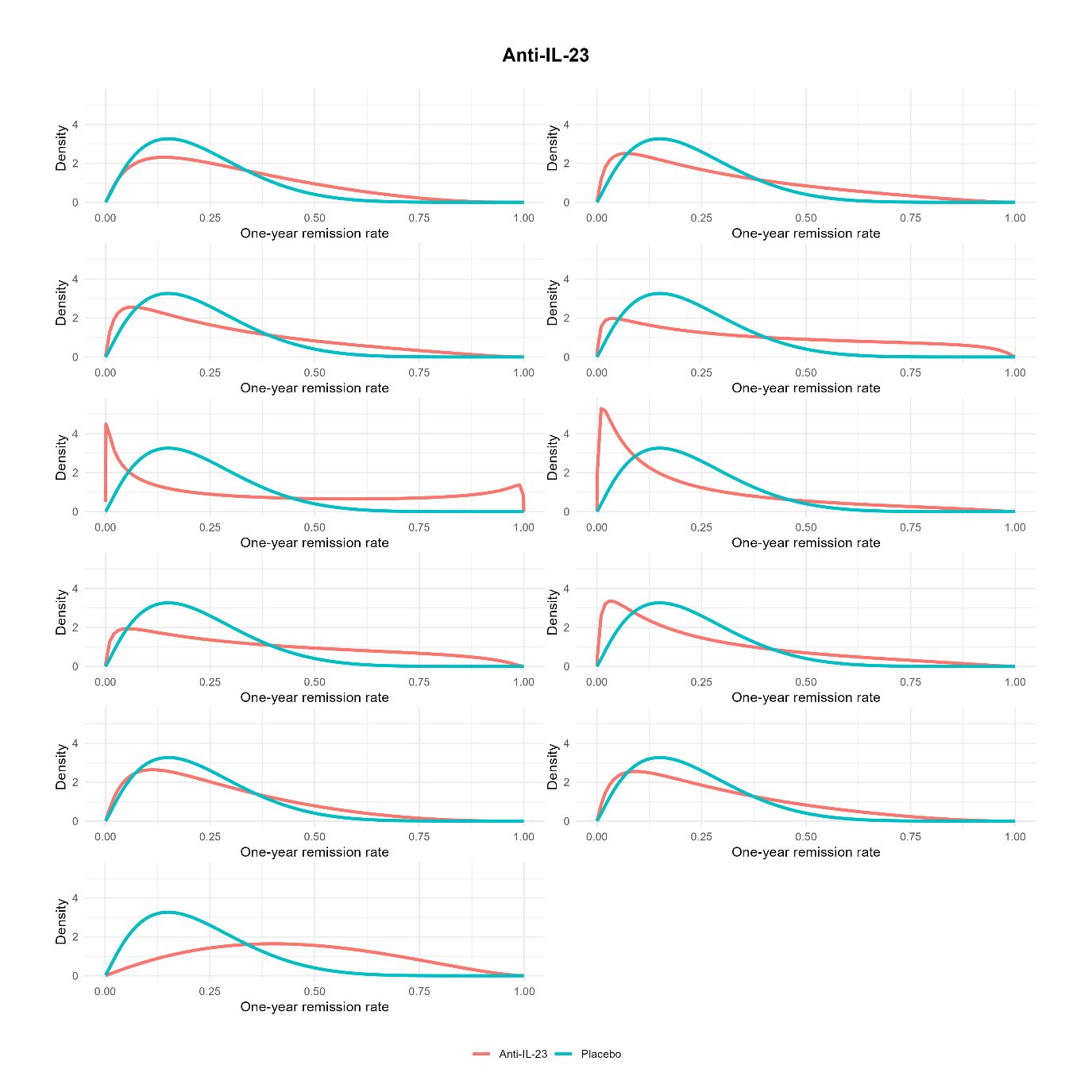
**

**Supplementary Figure 9. Effective sample size** **accounted for by an elicited prior depending on the standard error of the sample mean.**

**
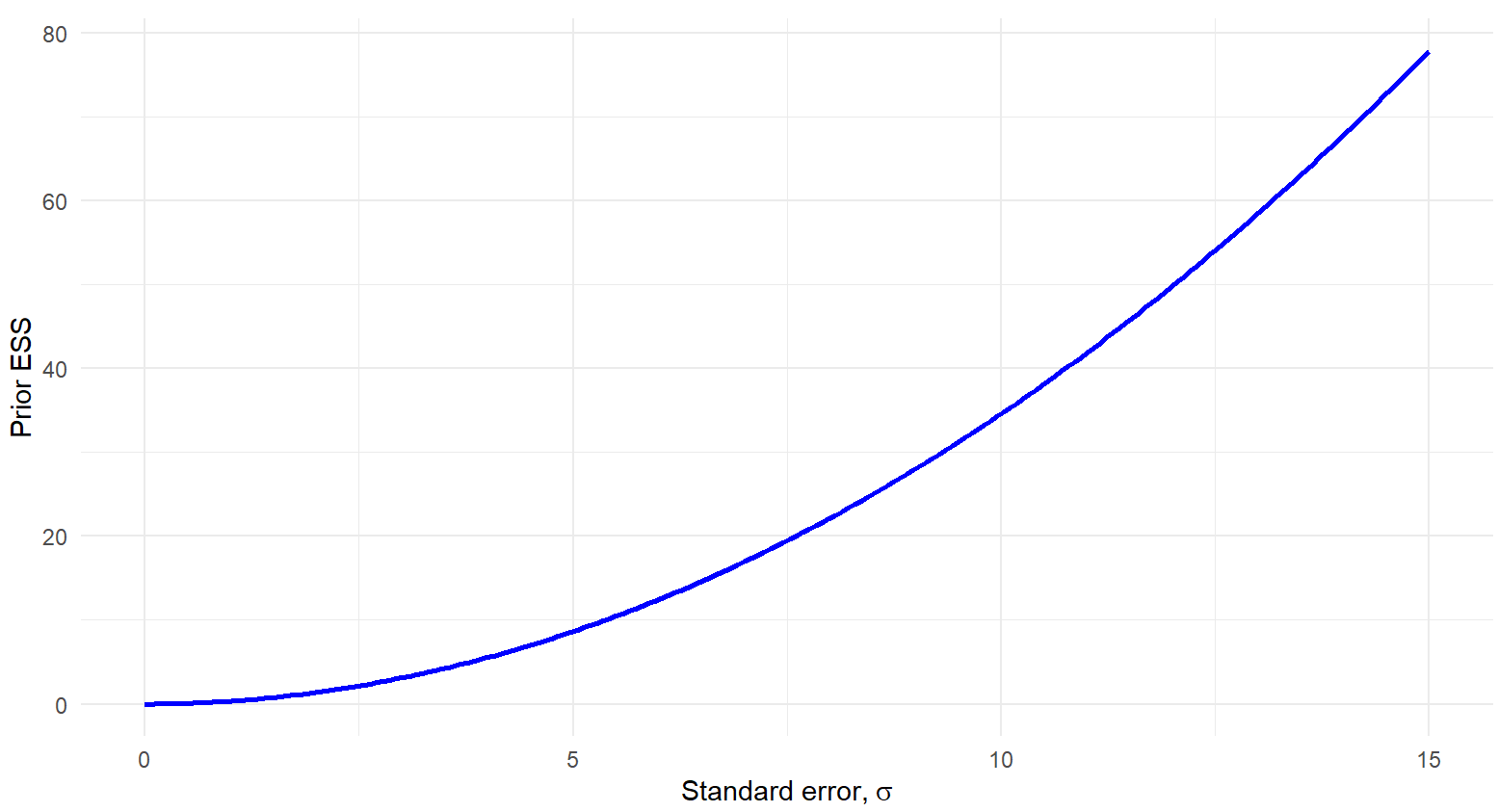
**
